# Patient-derived intrafemoral orthotopic xenografts of peripheral blood or bone marrow from acute myeloid and acute lymphoblastic leukemia patients: clinical characterization, methodology, and validation

**DOI:** 10.1007/s10238-022-00884-3

**Published:** 2022-09-19

**Authors:** Jun Li, Hongkui Chen, ShiZhu Zhao, Danyi Wen, Lintao Bi

**Affiliations:** 1 Department of Hematology and Oncology, China–Japan Union Hospital of Jilin University, No. 126 XianTai Street, Changchun, Jilin, 130033 China; 2 Shanghai LIDE Biotech, Co. Ltd, No. 77-78, Lane 887, Zuchongzhi Road, Pudong, Shanghai, China

**Keywords:** Acute leukemia, Patient-derived orthotopic xenograft (PDOX), Methodology, Peripheral blood, Bone marrow

## Abstract

Acute myeloid leukemia (AML) and acute lymphoblastic leukemia (ALL) are malignant clonal diseases of the hematopoietic system with an unsatisfactory overall prognosis. The main obstacle is the increased resistance of AML and ALL cells to chemotherapy. The development and validation of new therapeutic strategies for acute leukemia require preclinical models that accurately recapitulate the genetic, pathological, and clinical features of acute leukemia. A patient-derived orthotopic xenograft (PDOX) model is established using surgical orthotopic implantation. They closely resemble human tumor progression and microenvironment and are more reliable translational research tools than subcutaneous-transplant models. In this study, we established PDOX models by direct intrafemoral injection of bone marrow and peripheral blood cells from AML and ALL patients, characterized their pathology, cytology, and genetics, and compared the model's characteristics and drug responsiveness with those of the corresponding patients.

## Introduction

Leukemia is a common malignancy worldwide. Acute myeloid leukemia (AML) and acute lymphoblastic leukemia (ALL) are monoclonal neoplastic proliferations of myeloid precursors or lymphoid progenitor cells. Ongoing advances in treatment have significantly improved 5-year survival, but the overall prognosis of acute leukemia is not satisfactory [1]. About 50% of AML patients and 20% of ALL patients experience a relapse, which is the greatest challenge in the management of acute leukemia [2, 3]. The main obstacle in the treatment of acute leukemia is the increased resistance of AML and ALL cells to chemotherapy [4, 5]. The development and validation of novel therapeutic strategies for acute leukemia require preclinical models that accurately recapitulate the genetic, pathological, and clinical characteristics of AML and ALL.

Preclinical experimental models of acute leukemia are limited because they differ from human disease in genetic heterogeneity, pathological characteristics, and drug responsiveness [6]. Patient-derived xenograft (PDX) models are established by transplantation of patient tumor tissue or cells into immunocompromised mice, the strains of which were developed for PDXs including nude (nu), severe combined immunodeficient (SCID), non-obese diabetic (NOD), NOD-SCID, and NOD-SCID-IL2rγ^null^ (NSG) strains [7, 8]. In one of the first acute leukemia PDX studies, researchers transplanted AML cells into nude mice; however, due to an intact B cell and NK cell function, grafting of acute leukemic cells in nude mice remained poor. Then the development of acute leukemia mouse models in severe combine immunodeficient (SCID) mice was an important step forward. SCID mice are lack of functional mature T and B cells, but retain NK function. Thus, primary AML injected intraperitoneally or implanted under the kidney capsules showed improved engraftment rates in SCID mice, but intravenous injection still remained poor and unreliable. To overcome these limitations above, researchers began to transfer patient-derived leukemic cells directly into BM by intrafemoral injection, which was regarded as one kind of the patient-derived orthotopic xenograft (PDOX) models with implantation of tumor cells into the corresponding anatomical position in mice [9, 10]. Actually since 1990s of the last century, PDOX models have been developed using samples obtained at surgery for patients with solid tumors including colon cancer, pancreatic cancer, gastric cancer, breast cancer, lung cancer and many other cancers [11]. To further improve engraftment rates, mouse models with more severe immunodeficiency strains such as NSG mice were developed by combining the SCID background with the non-obese diabetic (NOD) strain which have no functional B or T cells and reduced NK cell and macrophage activity. They showed superior engraftment rate compared to SCID mice even when injecting fewer primary leukemic cells. Moreover, the morphologic, phenotypic, and genetic characteristics of the expanded AML specimens seemed mostly preserved [10].

This study investigated the usefulness of PDOX mouse models of AML and ALL for the study of drug sensitivity in NCG mice. NCG mice as a kind of more severe immune-deficient strain being fairly similar to NSG mice was established by CRISPR/Cas9 technology. *Prkdc (Protein kinase, DNA activated, catalytic polypeptide*) and *Il2rg (Common gamma chain receptor)* genes were knocked out on NOD/ShiltJGpt background. The genetic background of NOD/ShiltJGpt resulted in natural immunodeficiency, such as complement system and macrophage defects [12]. Meanwhile, the Sirpa on NOD/ShiltJGpt has high affinity with human CD47, making it more suitable for colonization of human grafts (e.g., tumors and human cells) than other strains [13]. Loss of *Prkdc* gene leads to the inability of V(D)J recombination to occur, resulting in the inability of *T* cells and *B* cells to mature. Il2rg is a common subunit of various interleukin cytokine receptors, and the inactivation of Il2rg leads to the loss of six different cytokine signaling pathways, resulting in NK cell defects [14]. We established models from patients with AML and ALL, characterized their pathological, cytological, and genetic features, and compared the features and drug responsiveness of the models with those of the corresponding patients.

## Materials and methods

### AML and ALL patient samples

Peripheral blood or bone marrow samples were obtained from AML and ALL patients at the China–Japan Union Hospital of Jilin University. The study was reviewed and approved by the Institutional Ethics Committee, and written informed consent for study enrollment and blood or bone marrow collections was obtained from all patients.

### Antibodies and other reagents

Antibodies (Abs) used for flow cytometry, PE mouse antihuman CD33 (Cat#: 555,450) and APC mouse antihuman CD45 (Cat#: 555,485) were purchased from BD Biosciences, PerCP/Cy5.5 antihuman CD19 (Cat#: 302,229) was purchased from Biolegend. Cytarabine (Cat#: BD8499), imatinib (Cat#: BD42606), and ibrutinib (Cat#:BD254580) were purchased from Bide Pharmatech (Shanghai, China). Epirubicin (Cat#: 56,390-09-1) was purchased from Shandong New Time Pharmaceutical Co., Ltd., Company (Shandong, China). Vincristine (Cat#: MB1298) was purchased from Melone Pharma (Dalian, China). Cladribine (Cat#:CSN10004) was purchased from CSNpharma (Shanghai, China).

### Animals

The immunodeficient NCG (NOD/ShiLtJ-Prkdc em26Cd52 Il2rg em26Cd22) mice used in this study were purchased from GemPharmatech Co., Ltd. Company (Nanjing, China) and maintained in pathogen-free conditions at Shanghai LIDE Biotechnology Co., Ltd., Company (Shanghai, China).

### Establishment of the PDOX models using peripheral blood or bone marrow

6- to 8-week-old NCG mice were injected intrafemorally with 1–2 × 10^6^ peripheral blood or bone marrow cells of AML or ALL patients after being anesthetized with a 1.25% avertin solution, respectively.

### Evaluation of the PDOX models and patient samples

#### Flow cytometry

**Fig. 1:**
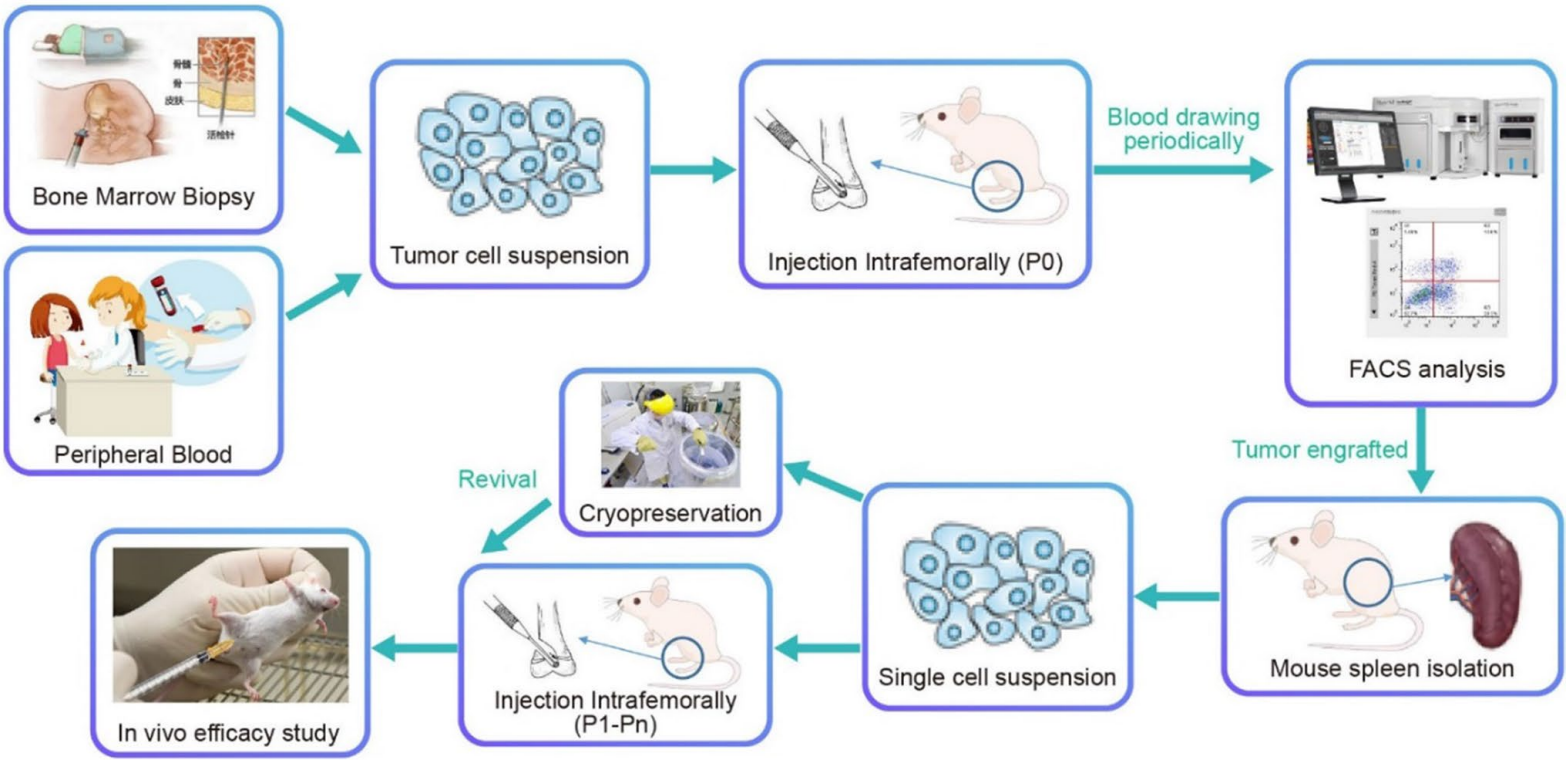
Procedures for PDOX establishment using AML/ALL samples

Engraftment of the orthotopic models was monitored by flow cytometry of peripheral blood from inoculated mice. The frequencies of CD45^+^CD33^+^ expression in AML and CD45^+^CD19^+^ expression for B-ALL were assayed. A 5–15% dual positivity indicated AML or ALL PDOX engraftment. The procedures of PDOX establishment are shown in Fig. 1. Staining and flow cytometry were performed following the manufacturers’ protocols. An Attune NxT flow cytometer was used with Attune NxT v4.2.0 software.

#### Additional analysis

The presence of gene mutations that commonly occur in AML and ALL patients was investigated by next generation sequencing of peripheral blood and bone marrow samples obtained from the AML and B-ALL patients and the xenografts in the PDOX mouse spleen samples. Chromosomal analysis of human AML and ALL cells in mouse spleen samples from the established PDOX models was performed by karyotyping and standard G-banding.

#### Standard of care in AML and ALL PDOX models

Cladribine, cytarabine, imtatinib, or epirubicin in combination with vincristine were used to validate the in vivo efficacy of standard chemotherapies in the AML and ALL PDOX models. Cladribine and imatinib was given daily via gavage at 0.4 mg/dose and 25 mg/kg, respectively. Cytarabine was given on days 1–5 by intraperitoneal injection at 10 mg/kg. Epirubicin was given weekly by intraperitoneal injection at 1 or 2.5 mg/kg. Vincristine was given weekly by intravenous injection at 0.5 mg/kg. The body weight of each animal was recorded every day, and doses were normalized against the individual weight to ensure consistency among groups. Flow cytometry was performed weekly to determine AML/ALL progression and the anti-tumor effectiveness of each treatment.

## Results

### Identification of patient samples

In the validation phase, we identified potential markers and targets in patient samples by next generation sequencing, G-banding, and flow cytometry, and the results are shown in Table 1.

**Table 1:** Cell surface markers, chromosome characteristics, and genetic analysis in leukemia patients

Model ID	FACS analysis	Chromosomal analysis	Gene mutation
LD1-0040-361,280	CD33^+^MPO^+^	No	NPMexon12A +
LD1-0040-362,349	CD34^+^CD117^+^CD13^+^CD33^+^CD38^+^	*t*(9;11)(p22;q23). The number is 10, which means the number of metaphase cells.	WT1exon7,c.1107A > G
			TP53exon4,c.215C > G
			ASXL1exon13,c.3759 T > C
			ASXL1_3'UTR,c.*22A > G
			TET2exon3,c.86C > G
			DNMT3Aexon2,c.27C > T
			DNMT3Aexon9,c.1122 + 7G > A
			DNMT3Aexon10,c.1266G > A
			DNMT3Aexon18,c.2173 + 26C > T
			DNMT3Aexon22,c.2597 + 30G > A
			MLL-AF9 fusion
LD1-0040-362,384	HLA-DR^+^CD13^+^CD15^+^CD33^+^MPO^+^	− 2, add(3)(p21), add(4)(q31), del(6)(q21q23), i(17)(q10), + mar. The number is 2, which means the number of metaphase cells/45, sl, − 10[5]. The Number is 5, which means the number of metaphase cells./46, sdl, + 18. The number is 3, which means the number of metaphase cells.	ND
LD1-0040-362,030	CD38^+^CD33^+^CD123^+^MPO^+^	Normal	ND
LD1-0040-361,780	CD13^+^CD33^+^CD56^+^MPO^+^CD117^+^	Normal	FLT3-ITD > 93 bp insertion
			NPM1A +
			DNMT3Aexon9,c.1122 + 7G > A
			DNMT3Aexon18,c.2173 + 26C > T
			DNMT3Aexon22,c.2597 + 30G > A
			TET2exon6,c.3647G > C
			TET2exon6,c.3655C > G
			TET2exon11,c.5162 T > G
			ASXL1exon12,c.3759 T > C
			ASXL1-3'UTR,c.*22A > G
			TP53exon2,c.74 + 14 T > C
			TP53exon4,c.97-29C > A
			TP53exon4,c.215C > G
LD1-0040-362,224	CD13^+^CD123^+^MPO^+^CD33^dim^CD34^dim^CD64^dim^CD117^dim^	ND	HOX11 +
			FLT3-ITD > 21 bp insertion
			NPM1A +
			DNMT3Aexon9,c.1122 + 7G > A
			DNMT3Aexon10,c.1266G > A
			TET2exon3,c.86C > G
			TET2exon3,c.2474delC
			TET2exon11,c.5082_5083insA
			ASXL1exon13,c.3758 T > C
			ASXL1-3'UTR,c.*22A > G
			WT1exon7,c.1107A > G
			TP53exon4,c.97-29C > A
LD1-0040-362,369	CD34^+^CD117^+^CD33^+^CD13^+^CD38^+^	*t*(7;11)(p15;p15). The number is 10, which means the number of metaphase cells.	DNMT3Aexon9,c.1122 + 7G > A
			DNMT3Aexon10,c.1266G > A
			DNMT3Aexon18,c.2173 + 26C > T
			DNMT3Aexon22,c.2597 + 30G > A
			DNMT3Aexon23,c.2674 T > A
			TET2exon11,c.5284A > G
			ASXL1exon13,c.3759 T > C
			ASXL1-3'UTR,c.*22A > G
			WT1exon7,c.1107A > G
LD1-0040-362,393	CD33^+^CD38^+^MPO^+^	Normal	HOX11 +
			NPM1A +
			DNMT3Aexon2,c.27C > T
			DNMT3Aexon9,c.1122 + 7G > A
			DNMT3Aexon10,c.1266G > A
			DNMT3Aexon18,c.2173 + 26C > T
			DNMT3Aexon22,c.2597 + 30G > A
			DNMT3Aexon23,c.2645G > A
			TET2exon3,c.3253_3256delACAA
			TET2exon6,c.3743 T > A
			TET2exon11,c.5284A > G
			TET2exon11,c.5412 T > C
			WT1exon7,c.1107A > G
			TP53exon4,c.215C > G
LD1-0040-362,499	CD117^+^CD33^+^CD123^+^CD56^dim^CD38^+^CD33^+^CD15^+^CD64^+^HLA-DR^+^CD36^+^CD11b^dim^CD13^dim^CD14^dim^CD9^dim^	Normal	KRASexon2,c.38G > A
			SRSF2exon1,c.284C > A
			TET2exon11,c.5540G > A
			NPM1exon11,c.860_863dupTCTG
LD1-0040-362,575	CD34^+^CD33^+^CD13^+^MPO^+^CD9^+^CD4^dim^CD11b^dim^HLA-DR^+^CD11c^+^CD38^+^CD14^+^CD123^+^CD64^+^CD2^+^CD36^+^	Normal	KITexon8,c.1251_1257 > GGGA
LD1-0041-362,073	CD34^+^HLA-DR^+^CD33^+^CD123^+^CD9^+^CD19^+^CD10^+^cCD79a^+^TDT^+^CD13^+^CD22^+^	+ 5, *t*(9;22), + der(22)t(9;22)	EP300exon6 > p.S507G
LD1-0041-362,356	CD45^dim^CD10^+^cCD22^+^CD19^+^CD33^+^	ND	JAK2exon16 > p.R683S
LD1-0041-362,478	CD45^dim^CD19^+^CD10^+^CD34^+^HLA-DR^+^CD9^+^CD24^+^CD58^+^CD38^+^cκ^dim^	Normal	ND
LD1-1041-362,519	CD38^+^HLA-DR^+^CD22^+^CD19^+^CD10^+^CD9^+^cCD79a^+^	del(1q), add(9p), del(9p), der(9), del(18q)	BRINP3exon7 > p.A369V
			CREBBPexon2 > p.A254T
			MED12exon8 > p.A412T
LD1-0041-362,021	CD19^+^CD22^+^CD34^+^CD38^+^cCD79a^+^	del(9)(p21). The number is 20, which means the number of metaphase cells.	ND

### Engraftment of AML and ALL PDOX model evaluated by fluorescence-activated cell sorting (FACS)

**Fig. 2:**
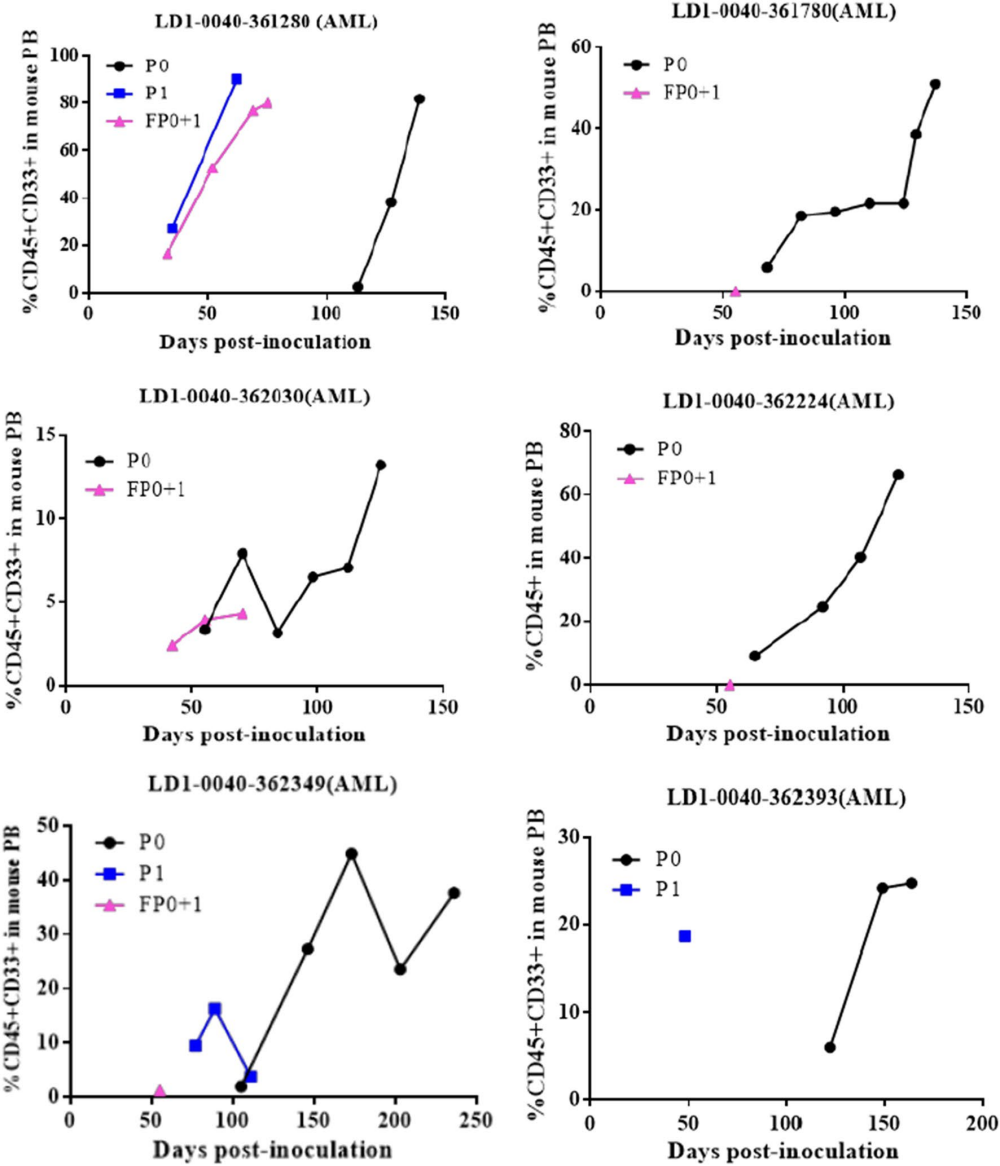

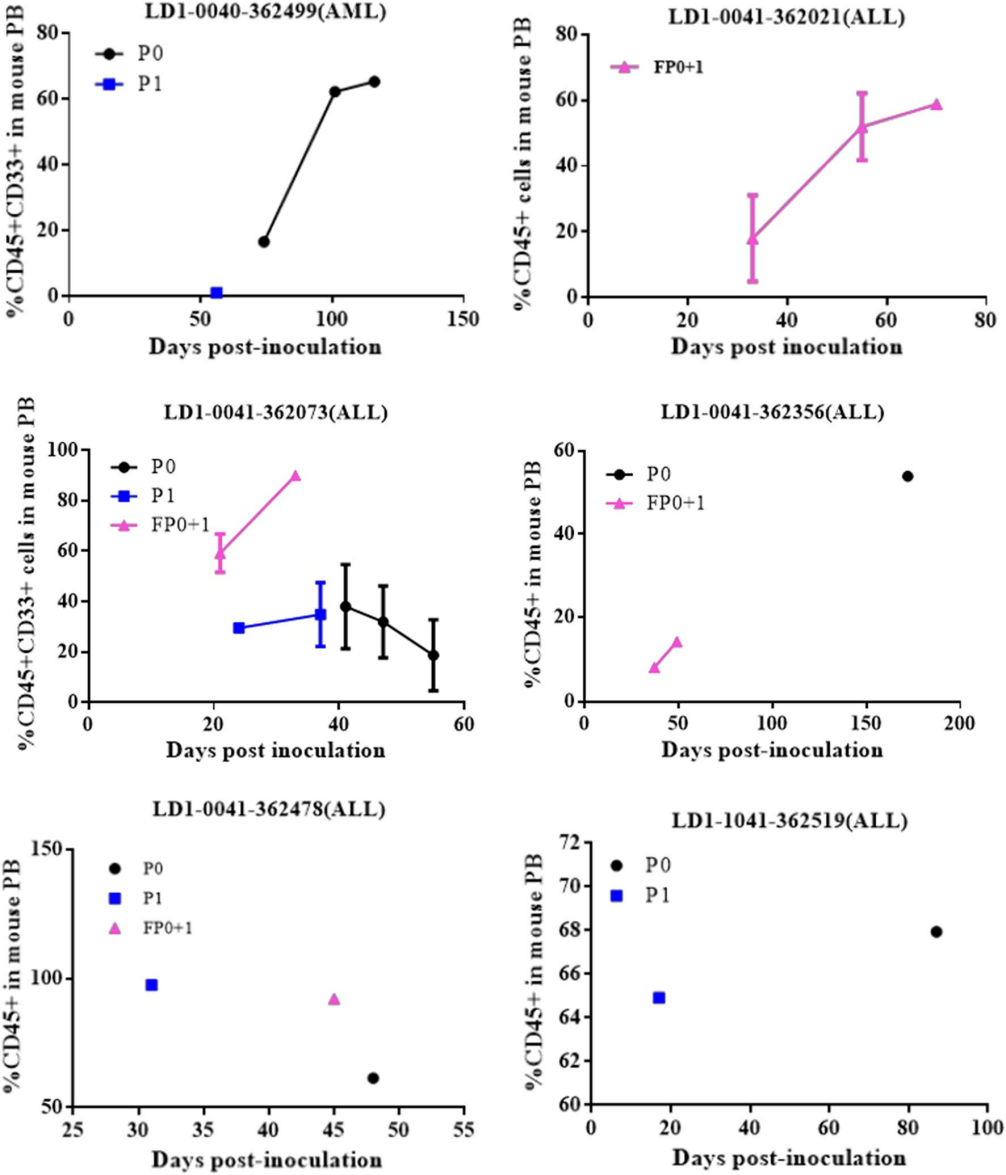
Establishment of PDOX Model in NCG mice. Cell suspensions prepared from patient samples were injected intrafemorally into NCG mice to generate the PDOX models. The frequency and percentage of CD45^ +^ or CD45 ^+^ CD33 ^+^ cells in mouse peripheral blood were determined by FACS at *P*0 (black lines), *P*1 (red lines), and FP0 + 1 (blue lines) of established PDOX models. Individual mice are shown as point values. Group results are shown with bars that give the standard deviation

Patient characteristics, diagnosis, and treatment history, are shown in Table 2. During this study, 67 samples of the peripheral blood (PB) or bone marrow (BM) of AML patients and 20 samples of the peripheral blood (PB) or bone marrow (BM) of ALL patients were processed for PDOX model establishment, which were prepared by intrafemoral injection of fresh blasts from the peripheral blood (PB) or bone marrow (BM) of AML and ALL patients into NCG mice and screened for their potential to initiate leukemia in the mouse models. The frequency of CD45^+^CD33^+^ cells or CD45^+^ cells in mouse peripheral blood was monitored weekly by FACS beginning 3–4 weeks after transplantation. To confirm the engraftment of human AML/ALL in the mouse models, P0 indicates the primary passage of patient cell suspension prepared from patient blood or bone marrow samples. When the percentage of positive cells reached 10%, single cell suspensions prepared from the mouse spleen were inoculated intrafemorally into naïve NCG mice (P1), or cryopreserved in liquid nitrogen until inoculation (FP0 + 1). The percentages of leukemia cells at P0, P1, and FP0 + 1 in 12 representative PDOX models are shown in Fig. 2. Successfully engraftment of PDOX models was defined as 10% CD45^+^CD33^+^ or CD45^+^ at P1. Successfully established PDOX required stable morphologic and molecular characteristics for at least two passages. In terms of our success tumor take rates, 10.45% (7/67) of AML patient samples and 40%(8/20) of ALL patient samples successfully initiated PDOX models. In addition, our study indicated that leukemia developed initially from the bone marrow of mice. After 5 weeks of transplantation in LD1-0024-361,280 AML PDOX model, the samples of the peripheral blood (PB) or bone marrow (BM) in 6 mice were collected at the same time for FACS detection. The results showed that the mean percentage of CD45^+^ CD33^+^ cells in the peripheral blood (PB) was 13.80% and the mean percentage of CD45^+^ CD33^+^ cells in bone marrow was 62.02%.

**Table 2:** Patient characterization

	Model ID	Sex	Age	Sample	Clinical diagnosis	Treatment history (sequentially)
AML	LD1-0040-361,280	Female	38	Bone Marrow	AML	Idarubicin + Cytarabine
						Cytarabine + Cladribine
	LD1-0040-362,349	Male	23	Bone Marrow	AML metastatic seminoma	Idarubicin + Cytarabine
						High-dose Cytarabine
	LD1-0040-362,384	Male	59	Peripheral blood	New-diagnosed AML	Azacitidine + Homoharringtonine + Cytarabine
						Idarubicin + Cytarabine
	LD1-0040-362,030	Female	56	Peripheral blood	New-diagnosed AML	Idarubicin + Cytarabine
						High-dose Cytarabine
						Homoharringtonine + Cytarabine
						Chidamide
						Anti-PD-1 ab
	LD1-0040-361,780	Male	49	Peripheral blood	New-diagnosed AML	Idarubicin + Cytarabine
						High-dose Cytarabine
						Azacitidine + Homoharringtonine + Cytarabine
						Azacitidine + Chidamide
						Etoposide + Cytarabine
	LD1-0040-362,224	Male	75	Bone Marrow	New-diagnosed AML	Hydroxycarbamide
	LD1-0040-362,369	Male	57	Bone Marrow	New-diagnosed AML	Idarubicin + Cytarabine
						High-dose Cytarabine
						Azacitidine
	LD1-0040-362,393	Female	57	Peripheral blood	New-diagnosed AML	Idarubicin + Cytarabine
						Idarubicin + High-dose Cytarabine
						Cytarabine + Azacitidine + Homoharringtonine
	LD1-0040-362,499	Male	68	Peripheral blood	New-diagnosed AML	N/A
	LD1-0040-362,575	Female	31	Bone Marrow	New-diagnosed AML	Idarubicin + Cytarabine
						High-dose Cytarabine
ALL	LD1-0041-362,073	Male	64	Bone Marrow	Recurrent ALL	CVDP
						Imatinib
						Cytarabine
	LD1-0041-362,356	Male	53	Bone Marrow	B-ALL	N/A
	LD1-0041-362,478	Male	15	Bone Marrow	ALL	CVDLP
						Cytarabine
						MTX
	LD1-1041-362,519	Female	38	Bone Marrow	Recurrent ALL	CVDLP
						Ibrutinib
	LD1-0041-362,021	Male	32	Bone Marrow	New-diagnosed ALL	N/A

Summarized clinical data of patients at the time of sample withdrawal. All samples used in this study were from patients with AML/ALL

Cell suspensions prepared from patient samples were injected intrafemorally into NCG mice to generate the PDOX models. The frequency and percentage of CD45 ^+^ or CD45 ^+ ^CD33 ^+ ^cells in mouse peripheral blood were determined by FACS at P0 (black lines), P1 (red lines), and FP0 + 1 (blue lines) of established PDOX models. Individual mice are shown as point values. Group results are shown with bars that give the standard deviation.

### Consistency of cell surface markers in patient samples and PDOX models

FACS analysis of cell surface markers in peripheral blood of patient samples and the PDOX models established using the samples are shown in Table 3. CD19 was positive in both patient samples and all four of the corresponding PDOX ALL models. The ALL were B cell-derived phenotypes. All four were CD10^+^ and CD38^+^, suggesting that CD19^+^CD10^+^CD38^+^ cell populations might be a useful as a panel to determine the establishment of PDOX in this type of B-ALL. The series of ALL-associated antigens revealed that the immunophenotype of PDOX cells was consistent with the primary specimens for most antigens tested and that they preserved the disease characteristics of the patients that they originated from.

**Table 3:** Consistency of partial PDOX with the clinical: FACS analysis

Model ID	Clinical		PDOX FACS	
	Immunophenotype	Result	Immunophenotype	Result
LD1-0041-362,073	Abnormal cells	69.44%	Abnormal cells	86.77%
	CD34	+	CD34	+
	HLA-DR	+	HLA-DR	Not detected
	CD33	+	CD33	Not detected
	CD123	+	CD123	+
	CD9	+	CD9	Not detected
	CD19	+	CD19	+
	CD10	+	CD10	+
	cCD79a	+	cCD79a	Not detected
	TDT	+	TDT	+
	CD13	+	CD13	Not detected
	CD22	+	CD22	Not detected
			CD24	+
			CD81dim	+
			CD73	+
	CD20	−	CD20	−
	CD38	−	CD38	−
LD1-1041-362,519	Abnormal cells	90.83%	Abnormal cells	94.74%
	CD38	+	CD38	+
	HLA-DR	+	HLA-DR	Not detected
	CD22	+	CD22	Not detected
	CD19	+	CD19	+
	CD10	+	CD10	+
	CD9	+	CD9	Not detected
	cCD79a	+	cCD79a	Not detected
	CD34	+ / −	CD34	−
	TDT	+ / −	TDT	Not detected
			CD81	+
	CD20	−	CD20	−
	CD123	−	CD123	−
LD1-0041-362,021	Abnormal cells	94.40%	Abnormal cells	80.25%
	CD19	+	CD19	+
	CD22	+	CD22	No detection
	CD34	+	CD34	+
	CD38	+	CD38	+
	cCD79a	+	cCD79a	No detection
	CD10	−	CD10dim	+
	cIgM	−	cIgM	No detection
			CD24	+
			CD81dim	+
			CD123	+
	CD20	−	CD20	−
			CD73	−
LD1-0041-362,478	Abnormal cells	84.98%	Abnormal cells	98.44%
	CD19	+	CD19	+
	CD10	+	CD10	+
	CD34	+	CD34	−
	HLA-DR	+	HLA-DR	No detection
	CD9	+	CD9	No detection
	CD24	+	CD24	No detection
	CD58	+	CD58	No detection
	CD38	+	CD38	+
	cκdim	+	cκdim	No detection
			CD81	+
			CD20	−
			CD123	−

” + ” means positive,”-” means negative

### Chromosome analysis of clinical samples and PDOX cells

**Fig. 3:**
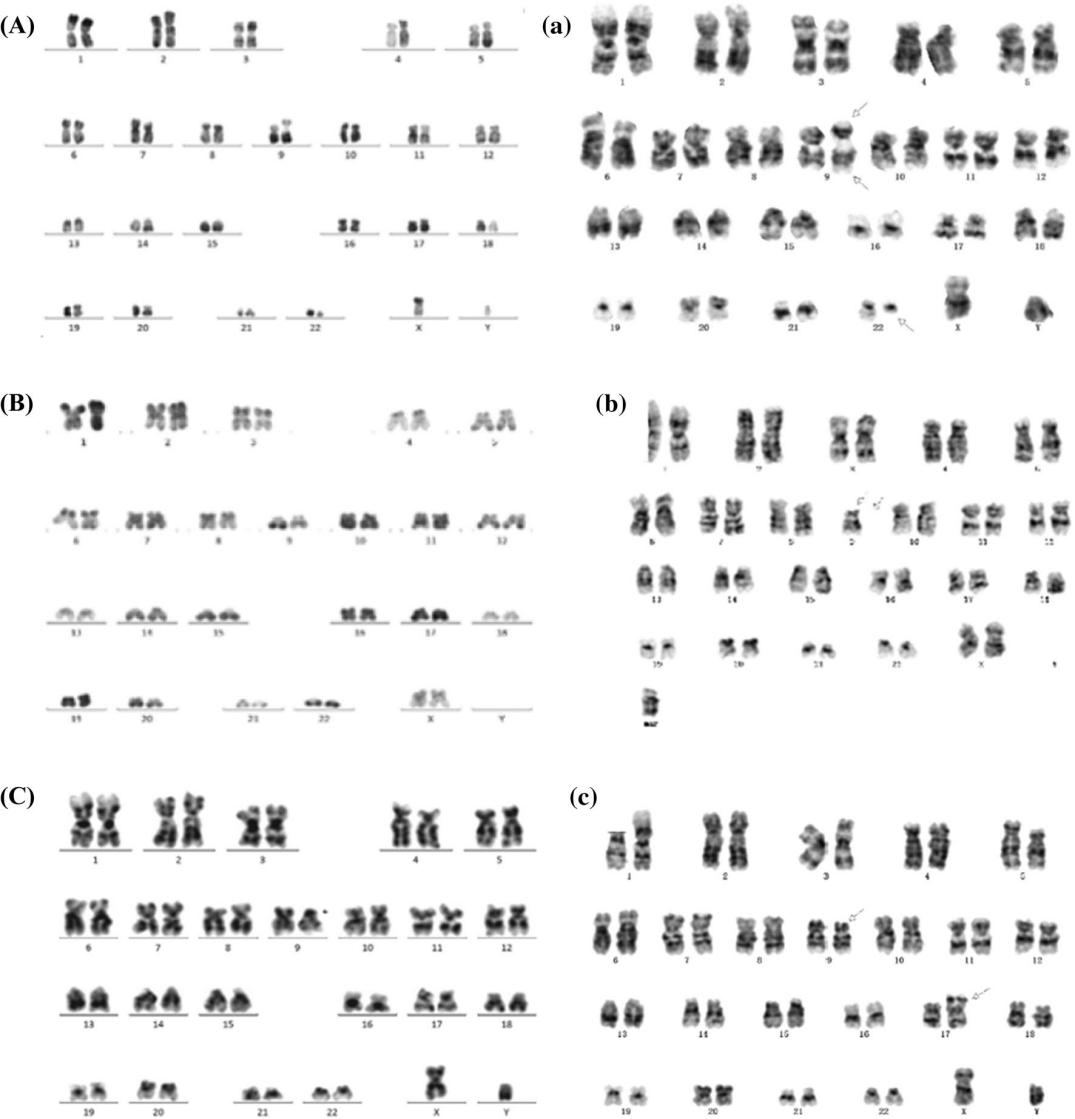
Karyotypes **A** of peripheral blood from an ALL patient (model ID: LD1-0041-362,073) and of **a** mouse peripheral blood from the LD1-0041-362,073 PDOX model. **B** Karyotypes of bone marrow from an ALL patient (model ID: LD1-1041-362,519) and of **b** mouse bone marrow from the LD1-1041-362,519 PDOX model. **C** Karyotypes of bone marrow of an ALL patient (model ID: LD1-0041-362,021) and **c** mouse bone marrow of the LD1-0041-362,021 PDOX model

G-banding karyotype analysis showed normal karyotypes and revealed that the karyotypes of PDX cells of LD1-0041-362,073 and LD1-0041-362,021 were similar to that of patient specimens. However, LD1-1041-362,519 showed a different karyotype in the PDX versus patient, the discordance between cytogenetic result of clinical specimen and PDOX on 1q21 and 18q21 may result from clonal selection. Moreover, because there is a complex structural variation on chromosome #9, it is uncertain whether it is a discordance (may also result from clonal selection) or different resolution, it would be better to have more clinical data to verify this. (Table 4, Fig. 3).

**Table 4:** Consistency of partial PDX/PDOX with the clinical: Chromosomal analysis

Model ID	Tissue type	Clinical specimen	PDOX result
LD1-0041-362,073	peripheral blood	+ 5	
		*t*(9;22)(q34;q11.2)[18]. The number is 18, which means the number of metaphase cells/51	t(9;22)(q34;q11.2)
		+ der(22)*t*(9;22)(q34;q11.2)[1]. The number is 1, which means the number of metaphase cells/48	der(22)t(9;22)[cp14]/46
LD1-1041-362,519	Bone marrow	del(9)(p13)	Normalization
		der(9)[18]. The number is 18, which means the number of metaphase cells/46	− 9
		add(9)(p13)	del(9)(p22)
		del(1)(q21)	Normalization
		del(18)(q21)	Normalization
LD1-0041-362,021	Bone marrow	Normalization	Normalization
		Normalization	del(9)(p12p21)
		del(9)(p21). The number is 20, which means the number of metaphase cells.	*i*(17)(q10)[20]. The number is 20, which means the number of metaphase cells.

### PDOX and the parental clinical sample have similar genetic profiles

An AML patient (LD1-0040-361,280) had a mutation on exon 12 of NPM1, a typical hot-spot alteration seen in AML patients without chromosome abnormalities [13]. As shown in Table 5, an NPM1exon12A mutation was found in the PDOX model by either RNAseq or whole exon sequencing. Other mutations such as c-kit/D816V, CEBPA, or FLT3/ITD that are often found in AML were not present in either the PDOX model or the original patient sample. The result indicates consistency in the genetic profiles of the patient and the PDOX model.

**Table 5:** Consistency of partial PDX/PDOX with the clinical: Genetic alteration

Model ID	Gene	Clinical result	PDOX result				
			WES_Mut(AF)	RNA_Mut(AF)	RNA_TPM	RNA_TPM_zscore_GTEx	RNA_TPM_zscore_TCGA
LD1-0040-361,280	c-kit/D816V	–	–	–	12.5	4.200862	− 1.08643
	CEBPA	–	–	–	117.22	1.922675	0.460406
	NPM1exon12A	+	Trp288fs(0.312)	Trp288fs(0.355)	1558.02	4.793047	3.388323
	FLT3/ITD	–	–	–	146.78	2.790451	0.758332

### Standard of care validation with therapeutic regimens used in clinical practice

**Fig. 4:**
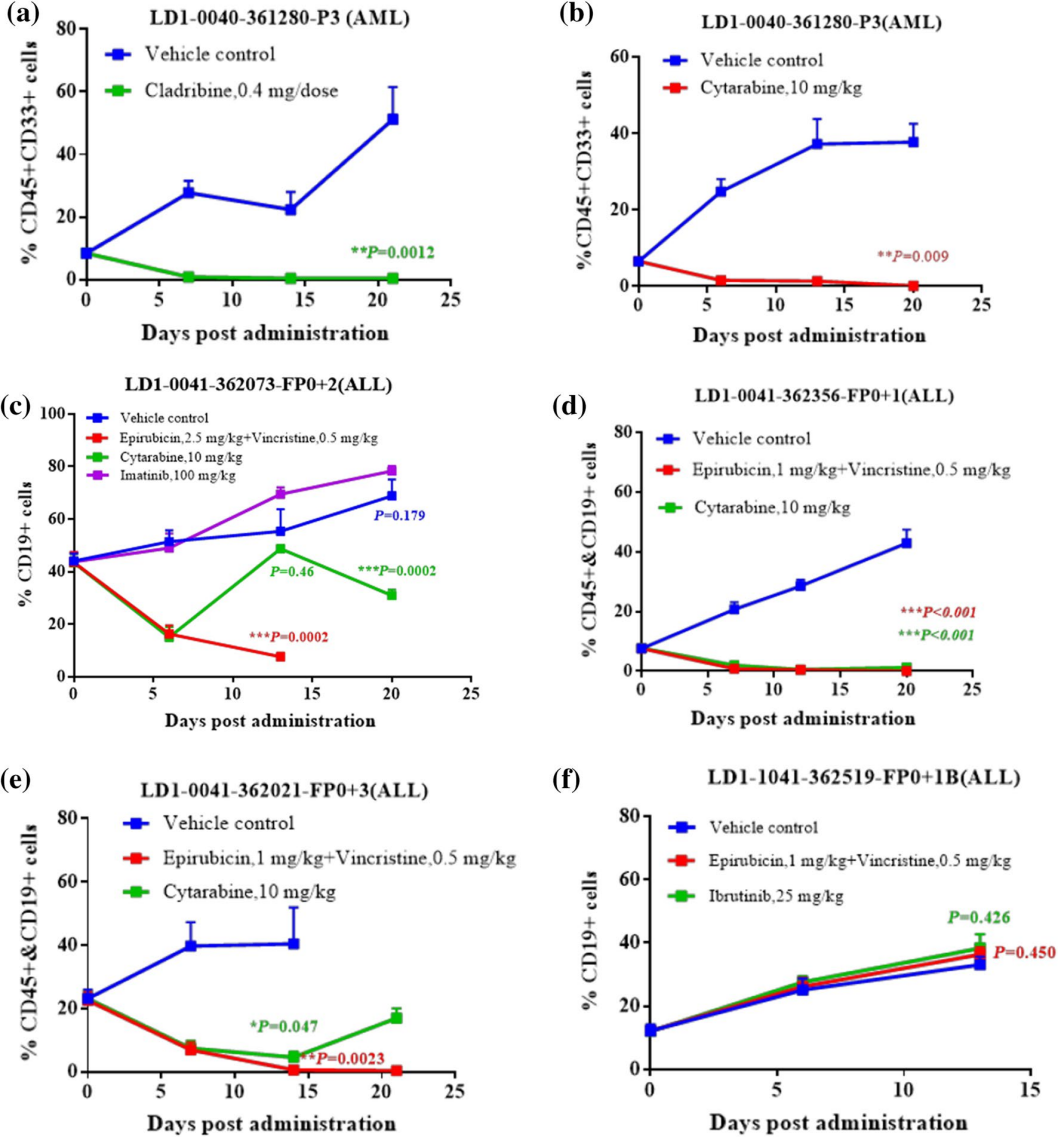
Standard of care validation in PDOX models. AML PDOX (LD1-0040-361,280) and ALL PDOXs (LD1-0040-362,073, LD1-0040-362,356, LD1-0040-362,021, and LD1-0040-362,519) models were used for standard of care validation. Treatment of the model mice began after randomization to the indicated regimen. Cell surface markers (CD45^+^, CD33^+^, and/or CD19^+^) were monitored weekly until the end of the study. Data are means ± standard deviation and *p* < 0.05 indicates statistical significance versus control

Validation using the same treatments that were administered to patient was performed in five of the established PDOXs. Cytarabine, which is commonly given to AML/ALL, patents had anti-tumor activity in both the AML and the ALL PDOXs (Fig. 4B–E). In the AML PDOX, cladribine completely eliminated the tumor (Fig. 4A). Epirubicin and vincristine, which are components in CEOP treatment [14] had good anti-tumor activity in three of the four ALL PDOX models (Fig. 4C–E), but not in the LD1-0040-362,519 model (Fig. 4F). Imatinib and ibrutinib, two small molecules that target BCR-Abl and BTK, respectively, were not effective in all the PDOX models (Fig. 4C and F). As shown in Table 2, response or non-response of the PDOX models was consistent with the clinical outcomes that occurred in response to those agents by the corresponding patients. The patient responses to treatment were maintained in the PDOX mouse models.

AML PDOX (LD1-0040-361,280) and ALL PDOXs (LD1-0040-362,073, LD1-0040-362,356, LD1-0040-362,021, and LD1-0040-362,519) models were used for standard of care validation. Treatment of the model mice began after randomization to the indicated regimen. Cell surface markers (CD45^+^, CD33^+^, and/or CD19^+^) were monitored weekly until the end of the study. Data are means ± standard deviation and *p* < 0.05 indicates statistical significance vs. control.

## Discussion

Patient-derived xenografts(PDX) are based on transferring primary tumors directly from the patient into an immunodeficient mouse. There are several advantages with PDXs including retaining the histological characteristics of patient tumors, such as tumor heterogeneity and tumor microenvironment [15]. When compared with traditional subcutaneous-transplant PDX model, PDOX models allowed the evaluation of tumor behavior in an organ-specific microenvironment. Furthermore, the PDOX models have been shown to have advantages over subcutaneous-transplant models, particularly with metastasis [11, 16]. AML and ALL PDOX mouse models were established by intrafemoral injection of leukemia cells from patients into triple immunodeficient NCG mice. The xenografted mouse models faithfully mimicked the pathology, cytology, karyotype, and genetic profile of the corresponding patient samples. The responsiveness of the AML and ALL PDOX mouse models to chemotherapy drugs was highly consistent with the clinical outcomes of the corresponding patients. The findings suggest that the AML and ALL PDOX mouse models established in this study preserved the features of the disease in the patients.

Heterogeneity of the malignant cells, with multiple mutant clones and subclones is a hallmark of acute leukemia [17, 18]. Tumor cell heterogeneity in acute leukemia reduces the effectiveness of treatment [19]. Culturing and passaging exert a selective pressure on leukemia cells that favors the survival of undifferentiated cells and results in loss of original tumor cell heterogeneity [20]. Therefore, the behavior of leukemia cell lines is profoundly different from the patient’s original tumor. PDX models of AML and ALL, which better represent the genetic and phenotypic heterogeneity of the corresponding leukemia, are indispensable for translational studies in acute leukemia [21, 22]. Establishment of PDX models of hematological malignancies remains challenging because leukemia cells have low take rate when transplanted into mice. The low success rate might be the result residual natural killer (NK) cell activity in non-obese diabetic (NOD)/severe combined immunodeficient (SCID) mice [23–25]. Crossbreeding NOD/SCID and interleukin (IL)-2 receptor *γ*-deficient mice has produced triple immunodeficient NCG, NSG, or NOG mice that lack functional *T*, *B*, and NK cells and are available from a number of suppliers [26]. The use of the triple immunodeficient NSG and NOG mouse models has significantly improved the efficiency of ALL and AML tumor engraftment in mouse models [27–29]. Triple immunodeficient NCG mice are not yet widely used to generate PDX models. This study demonstrated that NCG mice are also a kind of ideal models for the establishment of AML and ALL PDX models.

The implantation site of cancer cells affects the growth characteristics and phenotype of the PDX model [30]. Early PDX models of leukemia were established by heterotopically implanting cancer cells into the subcutaneous or intravenous space [9, 17, 23], and patient-derived heterotopic xenografts are widely used in cancer research. In this study, leukemia cells were injected intrafemorally into NCG mice to generate PDOXs. It is technically easier to perform and monitor tumor size of heterotopic implantation than establishing PDOXs [21]. Establishment of the leukemia PDOX model is time-consuming and technically challenging. The main advantage of PDOX models is a preserved tumor microenvironment and better modeling of tumor metastasis than heterotopic implantation [18].

An ideal research model should recapitulate the phenotypic and genetic characteristics of the original tumor in patients. In this study, the pathology of the mouse spleen and the surface markers of the engrafted cells from the PDOX models faithfully resembled the phenotypes of the patient leukemia samples. The karyotypes of PDOX cells were also highly consistent with those of the patient samples. We also compared the genetic profiles of PDOX models, and the clinical samples used for grafting by next generation sequencing. The *NPM1* gene is the most frequently mutated gene in AML. We found that the *NPM1* gene mutation in the PDOX model was consistent with the patient’s bone marrow sample, suggesting the PDOX model has stable genetic profiling.

In conclusion, it is feasible to establish AML and ALL PDOX models using the triple immunodeficient NCG mouse model. The established PDOX models preserve the pathological, phenotypic, and genetic characteristics of the clinical samples, and they also have similar drug sensitivity to the corresponding patients. The PDOX mouse model of acute leukemia provides a clinically relevant platform for testing novel chemotherapy drugs. Ultimately, the PDOX mouse model may be used for developing precision medicine approaches to treat leukemia.
